# Advances in Targeting HER3 as an Anticancer Therapy

**DOI:** 10.1155/2012/817304

**Published:** 2012-11-07

**Authors:** Ning Jiang, Nabil F. Saba, Zhuo Georgia Chen

**Affiliations:** ^1^State Key Laboratory of Oncology in Southern China, Department of Radiation Oncology, Sun Yat-Sen University Cancer Center, 651 Dongfeng Road East, Guangdong, Guangzhou 510060, China; ^2^Department of Hematology and Medical Oncology, Winship Cancer Institute, Emory University School of Medicine, 1365-C Clifton Road, NE, Atlanta, GA 30322, USA

## Abstract

HER3 (ErbB3) is a unique member of the human epidermal growth factor receptor (EGFR) family (ErbB family). It functions only through dimerization with other members of the ErbB family and modulates activity and sensitivity to targeted cancer therapies. This paper briefly describes the mechanism of HER3 in signal transduction and its potential role in acquired resistance to EGFR- and HER2-targeted therapies. We also consider recent developments in HER3-targeting therapeutics and their combination with inhibitors of other ErbB members in clinical applications.

## 1. Introduction

**Figure 1:**
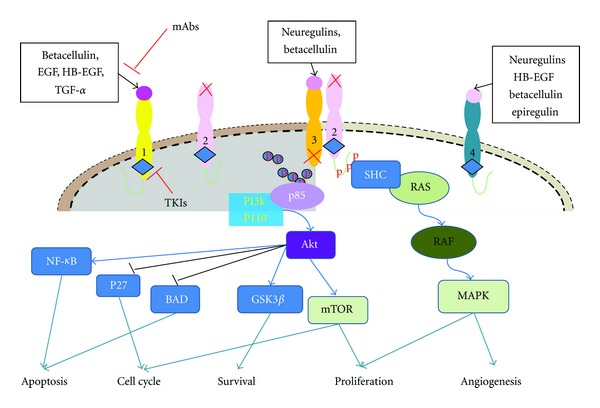
General features of the HER family. EGFR, HER3, HER4 have intact ligand binding sites. HER2 fails to bind any known ErbB ligands and HER3 has impaired catalytic activity. Two main strategies to target HER receptors for cancer treatment include monoclonal antibody (mAb) and tyrosine kinase inhibitor (TKI) approaches. The HER2-HER3 heterodimer is considered the most potent HER pair as an oncogenic unit and is illustrated as a representative dimer. Two key signaling pathways activated by the HER family dimers are the MAPK pathway and the PI3 K/Akt pathway. Activation of HER3 leads to transcription of genes that drive cell proliferation, migration, differentiation and angiogenesis [12, 15].

HER3 is one member of the human epidermal growth factor receptor (EGFR) family which consists of four types of transmembrane tyrosine kinase receptors, HER1 (EGFR, ErbB1), HER2 (Neu, ErbB2), HER3 (ErbB3), and HER4 (ErbB4) (Figure 1). The general structure of ErbB members includes an extracellular ligand-binding region, an *α*-helical transmembrane segment, a cytoplasmic tyrosine-kinase-containing domain, and a C-terminal phosphorylation tail [1, 2]. ErbB members are widely expressed in epithelial, mesenchymal, and neuronal tissues and regulate cell division, proliferation, differentiation, and other normal cellular processes [3, 4]. These membrane receptors receive extracellular signals from their ligands including those preferentially binding to EGFR such as epidermal growth factor (EGF), epiregulin, betacellulin, transforming growth factor-*α* (TGF-*α*), as well as neuregulins which only bind to HER3 and HER4 [5, 6]. Their normal physiological expression and function are controlled by the spatial and temporal expression of these ligands. Ligand binding triggers intracellular signaling through the formation of heterodimers or homodimers between ErbB receptors. Two key signaling pathways activated by the ErbB family are the RAS/RAF/MAPK pathway, which stimulates proliferation, and the PI3 K/Akt pathway, which promotes tumor cell survival [7]. As a result, the recruitment of intracellular signaling molecules and activation of a tightly controlled array of signaling pathways drive and regulate cell proliferation, and organ development and repair [1, 6]. 

HER3, which is the topic of our paper, is a unique member of the ErbB receptor family. Unlike EGFR and HER2, it cannot form a homodimer and lacks the intracellular kinase activity [8]. Although a recent study reported a weak tyrosine kinase activity, the prevailing view of HER3 is as an inactive “pseudokinase” [9], since its tyrosine kinase activity is ~1000 times weaker than EGFR [10]. However, the C-terminal region of HER3 contains six consensus phosphotyrosine sites which bind the SH2 domain of PI3 K, implicating its crucial role in the activation of the PI3 K/Akt pathway [11, 12]. It seems clear that activation of PI(3)K/Akt signaling by HER3 is able to overcome EGFR-targeted inhibition. Specifically, the redistribution of signaling functions to different ErbB family members allows HER3 to restore signaling activity despite significant inhibition of other ErbB kinases [13]. This unique capability of HER3 is not noted in EGFR, HER2, and HER4. Recent studies showed that expression and translocation of HER3 from the nucleus to the membrane were also responsible for resistance to EGFR or HER2 targeted therapy [13, 14]. This paper will focus on the function of HER3 in cancer development, its role in resistance to other ErbB targeted therapies, and its potential therapeutic value in the treatment of malignancies.

## 2. HER3 Structure and Expression in Human Cancers

HER3 was first identified in 1989 by Kraus et al. [16]. It maps to human chromosome 12q13 and translated into a protein showing homology to EGFR and HER2 in the extracellular ligand-binding domain and tyrosine kinase domain [16]. The ligand-binding domain of HER3 can be divided into four subdomains (I–IV), including two cysteine-rich regions (II and IV) and two flanking domains (I and III) that may define specificity for ligand binding [17]. HER3 shares 40–50% identity with EGFR and 40–45% with HER2 in each of these subdomains [18, 19]. The kinase domain of HER3 shares 60% and 62% similarity with EGFR and HER2, respectively [20]. However, both EGFR and HER2 have 83% amino acid sequence identity in their kinase domains, suggesting they are more closely related to each other than they are to HER3 [20]. A sequence comparison of the protein kinases reveals certain residues such as Cys-721, His-740, and Asn-815 have nonconservative substitutions in HER3. These changes diminish the catalytic activity of HER3 in its tyrosine kinase domain, indicating that HER3 may signal through an alternate biochemical response [20].

During organogenesis, HER3 expression and activation increase during postnatal maturation [21, 22]. HER3 knockdown mice exhibited severely underdeveloped sympathetic ganglia and partial lack of Schwann cells [23, 24], suggesting a unique function in the development of the fetal mouse brain. In human fetuses, HER3 transcripts were detected in the liver, kidney, and brain but not in heart or lung fibroblasts [16]. Similar to EGFR and HER2, HER3 mRNA was observed in normal keratinocyte and glandular epithelium tissues. However, unlike other family members, HER3 was not detectable in fibroblasts, skeletal muscle, and lymphoid cells [16]. This indicates that HER3 may have a specialized function in ectodermal development.

Upregulation of HER3 is commonly seen in various malignancies such as breast cancer, colorectal carcinoma, squamous cell carcinoma of the head and neck (SCCHN), uveal melanoma, and gastric, ovarian, prostate, and bladder cancers [25–28]. In human breast cancers both HER3 mRNA and protein are upregulated. Compared to normal breast tissue, HER3 protein overexpression has been reported in 50–70% of human breast cancers [29–31] and seems to be associated with metastasis [32], tumor size, and risk of local recurrence [33]. Increased HER3 mRNA or protein is commonly seen in tumors such as colon carcinomas and is associated with lymph node metastasis and a shorter time to progression [34–37]. In SCCHN, a high HER3 expression seems to be associated with increased metastasis and decreased overall survival [16, 38, 39]. Moreover, HER3 expression is correlated with resistance to the EGFR inhibitor gefitinib in SCCHN [40, 41]. This suggests that HER3 expression plays a significant role in carcinogenesis and would be a reasonable target for anticancer therapy. 

In addition to its cytoplasmic and membranous localization, HER3 protein has, like EGFR, been reported in cell nuclei [42, 43]. EGFR nuclear localization has been extensively studied and EGFR is suggested to function as a transcription factor, in chromatin remodeling as well as DNA repair [43]. Only a few studies have reported the nuclear localization of HER3, and these indicated similar functions. In immortalized human breast cells and breast cancer cells, HER3 was shown to have a mostly nuclear localization. However, after exposure to its ligand NRG1, a shift of HER3 from the nucleus to the cytoplasm was observed [44]. HER3 location seems to be specific to cancer type and stage. For example, the predominant pattern of HER3 staining in nonsmall cell lung cancer (NSCLC) is in the cytoplasm, but nuclear HER3 expression is highly associated with vascular and lymphatic invasion, which correlates with poor overall survival [45]. HER3 nuclear localization has also been reported in prostate cancer and more so in hormone refractory disease, and thus, is correlated with tumor progression [46]. In contrast, nuclear HER3 is independently correlated with favorable overall survival in uveal melanoma [47]. In SCCHN, HER3 was detected as either a cytoplasmic or a membranous dominant protein [39]. The membranous expression pattern is significantly prevalent in metastatic tissues and associated with worse overall survival [48]. These differences may be linked to the histological origin of cancer tissues. Understanding the underlying biology of different HER3 localization requires more studies. 

## 3. HER3 Signaling and Function in Cancer

Although HER3 lacks an innate kinase function and cannot form homodimers, it can still heterodimerize with other HER family members especially when signaling through the PI3 K/Akt pathway [3]. PI3 K/Akt lies at the hub of a plethora of downstream pathways and contributes to many biological processes critical for oncogenesis, including translation, survival, nutrient sensing, metabolic regulation, and cell cycle control. Dimer formation after ligand binding to HER3 results in C-terminal cross-phosphorylation between the dimer partners, creating docking sites that allow the recruitment and phosphorylation of downstream signaling components such as the p85 regulatory subunit of PI3 K [7]. Through binding to numerous proteins containing Src homology 2 domain or phosphotyrosine-binding (PTB) domain, different intracellular pathways are activated [49, 50]. Six consensus phosphotyrosine sites in HER3 can bind to the PI3 K p85 regulatory subunit and activate PI3 K/Akt downstream signaling [12, 51]. The HER3/PI3 K/Akt pathways have been implicated in breast, ovarian, colon, gastric, and lung cancer cells [52]. Studies of HER3 knockdown and inhibitors have established the importance of this pathway [53, 54]. 

A sequence analysis in the cytoplasmic domain of HER3 showed the binding sites for PI3 K, and also for GRB7, GRB2, SHC, and SRC [12]. Growth factor receptor bound 7 (GRB7) is an adapter molecule and plays a role in integrin signaling and cell migration. GRB7 interacts with HER3 mainly through its SH2 domain [52, 55]. GRB2 is not preferentially bound by HER3 and only interacts with HER3 when GRB7 is not present [12, 56]. Unlike GRB2/7 and SRC, SHC interacts with HER3 through the PTB domain rather than the SH2 domain. The SHC/HER3 interaction is essential for MAPK pathway activation [15, 57]. 

Because its functions are highly dependent on heterodimerization with other members, HER3 cannot transform cells through overexpression or mutational activation [58–60]. As an obligate partner with other family members, HER3 plays an important role in HER2 transforming and accelerating progression in human cancers. Transfection of HER3 into NIH3T3 fibroblast cells results in a low level of colony growth, but cotransfection with HER2 significantly enhanced the transformation effect compared with HER2 or HER3 alone [16, 43]. *In vivo* studies also showed that HER3 alone or in combination with EGFR was not tumorigenic, but cells transfected with HER3 and HER2 yielded xenograft tumors that grew more aggressively than other ErbB combinations and induced high levels of VEGF. Thus, the HER2-HER3 heterodimer is considered the most potent HER pair as an oncogenic unit.

HER3 seems to be as critical as HER2 for maintaining breast cancer cell proliferation [27]. Using different methods to knock down HER3 expression, inhibition of breast cancer cell growth was more potent than knocking down EGFR [27, 54]. Furthermore, preferential phosphorylation of HER3, but not EGFR, was observed in HER2-amplified breast cancer tissues [27]. However, in melanoma and pancreatic cancer, HER3 appears to be a preferred dimerization partner of EGFR. In experimental models, knockdown of HER3 reduces melanoma cell migration and invasion [61] whereas overexpression of HER3 significantly increased cell proliferation both *in vitro* and *in vivo* in pancreatic adenocarcinoma [62]. In SCCHN, HER3 membranous expression was found to be associated with decreased survival [48]. Elevated neuregulin-1 (NRG-1) and activation of that HER3 were enriched in a subset of SCCHN, suggesting HER3 might play a role in SCCHN [63].

## 4. Potential Role of HER3 in EGFR- and HER2-Targeted Therapies 

Because of their extensive overexpression in cancer tissues and important function in cancer progression, attempts to target ErbB family members in cancer therapy have been the focus of extensive research and have reached clinical applications in many cancers. Most drugs targeting the ErbB family are against EGFR and HER2 because the pro-oncogenesis function of these receptors is well understood [3, 64]. Two predominant types of ErbB-targeted drugs have been developed: monoclonal antibodies that target the extracellular domain, such as cetuximab and trastuzumab, and small molecule tyrosine kinase inhibitors (TKIs), such as gefitinib and erlotinib. 

Multiple antibodies targeting the EGFR have been approved for clinical use. Cetuximab, a monoclonal antibody targeting EGFR, is approved for treating SCCHN in combination with radiation therapy for locally advanced disease and in combination with platinum-based chemotherapy as a standard first line systemic therapy. In the landmark EXTREME trial, patients who received additional cetuximab had a significant reduction in the risk of death by 20% compared to patients receiving chemotherapy only and, for the first time for patients with recurrent metastatic SCCHN, the median survival was prolonged to 10.1 months [65]. In colorectal cancer, the efficacy of both monoclonal antibodies against EGFR, cetuximab and panitumumab, is dependent upon the mutational status of KRAS [66]. The EGFR-TKIs erlotinib and gefitinib have also shown dramatic effects against EGFR-mutant lung cancer and have been approved for second-line therapy in patients with metastatic NSCLC [67, 68].

HER2 amplification and overexpression have been reported in 18–25% of breast cancer, as well as in subsets of patients with gastric carcinoma, esophageal cancer, salivary gland tumor, and ovarian cancers [17, 18, 28, 69–71]. The humanized monoclonal anti-HER2 antibody trastuzumab (Herceptin; Genentech) is approved for use in breast cancer and has had a major impact in treating this disease [72]. More recently phase III Trastuzumab for Gastric Cancer (ToGA) trial showed that adding trastuzumab to chemotherapy significantly improves survival without negatively impacting quality of life in patients with advanced gastric or gastroesophageal junction cancer [73]. 

Despite these advances in therapy, mechanisms for EGFR resistance are documented and are the subject of intensive research, detailed in a separate paper published within this special issue. In lung cancer, although EGFR-TKI treatment leads to significant responses in patients with EGFR gene mutations, acquired resistance to these drugs inevitably occurs. Major described mechanisms of acquired resistance include KRAS/BRAF [74] and EGFR T790 M secondary mutation [74], amplification of the MET gene, as well as hepatocyte growth factor (HGF) expression [75] (detailed in a separate paper within the same issue). Targeted treatment of colorectal cancer has also been limited by resistance to anti-EGFR therapy. In a recommendation from the American Society of Clinical Oncology (ASCO) in 2009, patients with metastatic colorectal cancer in which codon 12 or 13 of the KRAS gene was found to be mutated were recommended not to receive anti-EGFR antibody therapy as part of their treatment [76]. Mechanisms of EGFR resistance continue to be a topic of interest in different tumor types including SCCHN where several possible mechanisms have been described including a deletion mutation of exon 2–7 of the extracellular ligand-binding domain of EGFR leading to a truncated form of EGFR (EGFR vIII) that is auto-phosphorylated in a ligand-independent way. EGFR vIII has been reported in close to 43% of SCCHN cases [77, 78]. 

**Figure 2:**
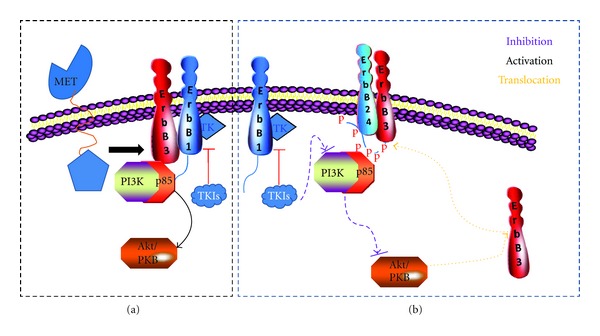
Mechanisms by which HER3 contributes to resistance to EGFR-targeted therapy. (a) The oncogenic receptor tyrosine kinase MET could phosphorylate HER3, leading to activation of the PI3 K/Akt pathway independent of EGFR kinase activity [57]. (b) Lengthy exposure of cancer cells to TKIs can lead to Akt downregulation which consequently increases HER3 translocation from the cytoplasm to the membrane through a feedback regulation. The phosphorylation of HER3 recruits PI3 K and further activates the PI3 K/Akt pathway which plays an important role in resistance to EGFR-targeted therapy [2].

Interestingly, resistance to EGFR-targeted therapy was claimed to be a family affair by several researchers [74]. In addition, compensatory HER3 signaling and sustained PI3 K/Akt activation have been implicated as playing an important role in the resistance to HER-targeted therapy [7, 13, 79, 80] (Figure 2). After lengthy exposure to inhibitors, cancer cells switched dependence between EGFR and HER2, but in both cases, HER3 was the common association partner [81, 82]. In HER2 dominant breast cancer cells, lengthy exposure to the EGFR inhibitors gefitinib or erlotinib or the HER2 inhibitor AG-825 led to the upregulation of HER3 and Akt phosphorylation in correlation with HER3 translocation from the nucleus to the membrane [13, 83]. The mechanism for increased expression of HER3 in the membrane features a resetting of the HER3 phosphorylation-dephosphorylation equilibrium, which is driven by Akt-mediated negative feedback signaling [13]. Increased HER3 ligand heregulin expression is also a possible mechanism of cetuximab resistance in colorectal cancer patients [84]. Furthermore, HER3 may work cooperatively with other receptor tyrosine kinases, such as hepatocyte growth factor receptor (HGFR; also known as MET) [83]. MET proto-oncogene amplification may be a cause of resistance to gefitinib. Phosphorylated HER3 was found to co-immunoprecipitate with PI3 K p85 unit in a MET kinase-dependent manner suggesting a role of HER3 in MET-induced resistance. [83]. It is worth noting that MET is one of the major signal transduction proteins which contribute to EGFR targeting resistance [85–87].

Notably, in SCCHN cell lines sensitive to the dual EGFR/HER2 reversible inhibitor lapatinib, elevated NRG1 and activated HER3 were strongly associated with lapatinib sensitivity [63]. At the same time, HER3 and HER2 expression were significantly associated with resistance to the EGFR inhibitor gefitinib but not cetuximab in SCCHN. The combination of gefitinib and the HER2-HER3 dimerization inhibitor pertuzumab provided additional growth inhibitory effect over gefitinib alone [88]. The mechanism through which HER3 functions as a biomarker for lapatinib sensitivity on the one hand, and a cause of gefitinib resistance on the other, is unclear but may be due to different activation mechanisms which need to be further uncovered. In breast cancer, EGFR and HER3 expression are substantially increased after long-term trastuzumab exposure [82]. In pancreatic cancer, HER3 is a preferred dimerization partner for EGFR and through the PI3 K/Akt pathway plays a role in modulating response to erlotinib. The siRNA-mediated inhibition of HER3 expression in pancreatic cancer cells resulted in an acquired resistance to erlotinib treatment. Hence, pancreatic cancer cells which lack HER3 become less critically dependent on EGFR signaling and, therefore, resistant to erlotinib [62]. In spite of the obvious evidence that high HER3 expression in pancreatic cancer cells and NSCLC cells confers increased sensitivity to gefitinib or erlotinib than in low HER3-expressing cells [89], the above resistance promoting function may be caused by an increase in the level of activated HER3 rather than of total HER3 protein expression.

## 5. Development of Agents That Target HER3

Both HER3 inhibitors and pan-ErbB inhibitors, which simultaneously inhibit HER3 and other family members, have been developed and a number of them are in early clinical development (Table 1). Most HER3 inhibitors under development target the extracellular domain of the protein. Schoeberl et al. used a computational model to explore the optimal way to therapeutically inhibit combinatorial ligands which induce activation of the HER3-PI3 K axis. This study revealed a dominant role of HER3 in Akt activation and suggested that targeting this key node of the ErbB signaling network might result in therapeutic benefits to cancer patients [90]. However, a principal technical challenge of targeting HER3 is that, unlike other HER family members, HER3 lacks enzymatic catalytic activity. This feature of HER3 indicates that its function cannot be inhibited by ATP bindingsite inhibitor TKIs.

MM-121 is a fully humanized anti-HER3 antibody that specifically blocks the binding of HRG1-*β* (a neuregulin-1 type I polypeptide) to HER3 [90]. Preclinical studies reported that pretreatment of pancreatic ductal adenocarcinoma cells with MM-121 followed by NRG-1 stimulation resulted in ligand-induced HER3 activation [91]. The combination of MM-121 with erlotinib completely abolished Akt activation in pancreatic cancer cells [91]. MM-121 also inhibited growth of ovarian cancer cells both *in vitro* and *in vivo *[90]. Gefitinib-resistant lung cancer cell lines were resensitized to gefitinib after being treated with MM-121 [92]. Meanwhile, MM-121 combined with the anti-EGFR antibody cetuximab showed a synergic effect in a lung cancer model [92]. At present, 9 clinical trials are recruiting patients for phase I or phase I-II study of MM-121 in combination with chemotherapy or other HER inhibitors such as cetuximab/gefitinib. These clinical trials will provide further evidence for the clinical use of MM-121 (data from http://www.clinicaltrials.gov/). MM-111 is a bispecific antibody, binding to two different target proteins, ErbB2 and ErbB3, and inhibiting the signaling downstream of these two cell receptors resulting in an inhibitory effect on the PI3 K pathway [93]. The safety and clinical activity of MM-111 is now under study in several phase I clinical trials.

Another HER3-targeted drug is U3-1287 (AMG888), the first fully humanized HER3 monoclonal antibody. This mAb inhibits proximal and distal HER signaling and induces rapid internalization of HER3 [94]. U3-1287 showed growth inhibition ability *in vitro* in multiple tumor cell lines such as breast, lung, and colorectal cancer and *in vivo* in pancreatic, NSCLC, and colorectal cancer xenograft models [95]. A phase I clinical trial of U3-1287 in the treatment of advanced solid tumors has been completed, the result of which has not been published yet (NCT00730470). Another ongoing phase Ib/II study found that this antibody was well tolerated in NSCLC patients when combined with erlotinib (NCT01222483) [94]. A phase Ib/II trial using U3-1287 in combination with trastuzumab and paclitaxel is currently recruiting patients with newly diagnosed metastatic breast cancer (data from http://www.clinicaltrials.gov/).

In addition to monoclonal antibodies, inhibition of HER3 dimerization with other ErbB family members is a valid approach. Pertuzumab targets the dimerization interface of HER2 thus disrupts ligand-induced HER2-HER3 dimerization. Several clinical trials showed a significant clinical benefit in HER2-positive breast cancer patients [3, 53, 96]. More evidence will be obtained from ongoing clinical studies using pertuzumab to treat other cancers such as gastric cancers, neuroendocrine tumors, and prostate cancers. 

The first-generation pan-ErbB inhibitor canertinib (CI-1033) inhibits all ErbB family members without affecting other tyrosine kinases. Several phase I-II clinical studies showed limited effect, preventing further clinical development of this drug [12, 52, 55]. Other multitarget inhibitors such as MEHD7945A, MP-470, and AZD8931 are still under clinical development.

## 6. Summary and Future Directions in HER3-Targeted Therapy

For decades, research around the ErbB family receptors has focused on the dysregulated catalytic kinase activities of EGFR and HER2. Recently, the important role of HER3 as an obligate partner for HER receptor dimerization and in primary and acquired resistance to HER2- or EGFR-targeted therapy has brought considerable attention to HER3 from cancer researchers. Unlike other members of ErbB family, HER3 has unique biological functions through its heterodimerization with other HER receptors. 

The increasing awareness of HER3 function in cancer progression and drug resistance has several implications for future research directions. First, due to the relationship of HER3 signaling with sensitivity or resistance to HER-targeted therapy, HER3 may be considered a valuable biomarker to monitor the efficacy of HER-targeted therapy. Secondly, the combination of HER3 and EGFR/HER2-targeted agents may be an efficient way to conquer drug resistance and, thus, enhance antitumor activity. Moreover, because the PI3 K/Akt pathway, which is downstream of HER3, plays an important role in cancer progression and drug resistance, it is reasonable to hypothesize that PI3 K/Akt inhibitors can also enhance the antitumor activity of HER-targeted drugs. Last, but not least, HER3 pathway inhibition can also be achieved by reducing the release of HER3 ligands, heregulin, and neuregulin.

In conclusion, HER3 is a focal point in HER family-induced cancer oncogenesis and, as such, constitutes a new potential biomarker and target for future cancer therapy.

## Figures and Tables

**Table 1:** HER3-targeted drugs under development.

Drug	Type	Target(s)	Development phase	Sponsor
MM-121	Humanized mAb	HER3	Phase I/II	Merrimack
U3-1287 (AMG 888)	Humanized mAb	HER3	Phase I	U3 Pharma GmbH
MM-111	Bispecific antibody	HER2-HER3	Phase I	Merrimack
Pertuzumab	Humanized mAb	HER2-HER3	Phase III	Genentech
MEHD7945A	mAb	HER1, HER3	Phase II	Genentech
MP-470 (Amuvatinib)	Pan inhibitor	HER1/2/3	Phase II	Astex Pharmaceuticals
AZD8931	Pan inhibitor	HER1/2/3	Phase I/II	AstraZeneca
